# Neuropsychological profile of Parkinson's disease patients selected for deep brain stimulation surgery

**DOI:** 10.1590/s1980-5764-2016dn1004007

**Published:** 2016

**Authors:** Flavia Amaral Machado, Carlos Roberto Rieder, Arlete Hilbig, Caroline Tozzi Reppold

**Affiliations:** 1 Universidade Federal de Ciências da Saude de Porto Alegre, Porto Alegre, RS Brazil.

**Keywords:** neuropsychology, Parkinson's disease, neurosurgery, deep brain stimulation

## Abstract

**Background:**

Parkinson's disease (PD) shows symptoms involving motor and non-motor complications, including cognitive and behavioral changes, such changes might to contraindicate deep brain stimulation surgery (DBS).

**Objective:**

The aim of study was to investigate the neuropsychological profile of patients with PD in a waiting list for DBS.

**Methods:**

The neuropsychological evaluation was held in 30 patients of the ISCMPA Movement Disorders Clinic, with surgical indication based on the criteria of the responsible neurologists, in the period of 12 months. Instruments used: MMSE, FAB, MoCA, BDI, Semantic Verbal Fluency, PDQ-39, PDSS; and the UPDRS and Hoehn-Yahr scale.

**Results:**

The patients were mostly male (66.7%) with a mean age of 59.37 (SD 10.60) and disease duration 9.33 (SD 4.08). There was cognitive impairment in 56.7% of patients by FAB and 76.7% by MoCA.

**Conclusion:**

Even in the earliest stages of the disease, there is the incidence of non-motor symptoms, especially in those subjects who had an early onset of the disease.

## Abstract (pt)

**Embasamento:**

A doença de Parkinson (DP) apresenta sintomas que envolvem complicações motoras e não-motoras, entre elas alterações cognitivas e comportamentais, tais alterações podem contraindicar a cirurgia de estimulação cerebral profunda (DBS).

**Objetivo:**

O objetivo de estudo foi verificar o perfil neuropsicológico dos pacientes com DP candidatos à DBS.

**Métodos:**

Realizou-se avaliação neuropsicológica em 30 pacientes do ambulatório de Distúrbios do Movimento da ISCMPA, com indicação cirúrgica segundo critério dos neurologistas responsáveis, no período de 12 meses. Os instrumentos utilizados: MEEM, FAB, MoCA, BDI, FV Semântica, PDQ-39, PDSS; e as escalas UPDRS e Hoehn-Yahr.

**Resultados:**

Os pacientes foram, em sua maioria, homens (66,7%), com média de idade de 59,37 (DP 10,6) e tempo de doença 9,33 (DP 4,0). Apresentaram comprometimento cognitivo em 56,7% dos pacientes segundo os escores do FAB e 76,7% segundo o MoCA.

**Conclusão:**

Mesmo em fases mais iniciais da doença, percebe-se a incidência de sintomas não motores, principalmente naqueles sujeitos que tiveram um início de doença precoce da doença.

## INTRODUCTION

Parkinson's disease is a movement disorder characterized by its main motor symptoms: bradykinesia, rigidity, resting tremor and postural instability.^1^ However, it is essential to emphasize that PD is considered a systemic disease and thus presents a variety of non-motor symptoms involving aspects such as memory loss, language/verbal fluency changes and executive function and visual-spatial ability deficits, among others. These cognitive and behavioral impairments negatively impact patient quality of life, can complicate treatment and represent signs of poor prognosis.^2^

Recommended treatments include surgery, in which one of the techniques used is deep brain stimulation (DBS), available through the Brazilian public health system and considered a good alternative in refractory cases. In addition to improving the cardinal motor symptoms of PD, DBS surgery reduces the complications that accompany dopaminergic therapies, such as motor fluctuations and dyskinesia.^3,4^ Indication is thorough and includes an established PD diagnosis for at least five years; absence of severe cognitive impairment or dementia; absence of other comorbidities; and response to preoperative levodopa, which is considered a good prognosis for postoperative outcomes.^5,6^

The purpose of this procedure is to implant a deep brain stimulator to control motor symptoms. This approach has shown good results in managing motor complications of the disease. However, the effects of DBS on non-motor symptoms (mainly cognition and psychological functioning) are inconsistent.^7^

Several publications have provided recommendations for surgical indication in PD ^5,7,8^ and debated the early indication of DBS to benefit patients before they develop non-motor complications, especially cognitive impairment that contraindicates the procedure. In addition, some studies have assessed the non-motor areas involved in PD, where the most frequent cognitive aspects are executive function, memory, language, and visuo-spatial skills.^2,4,9^ These symptoms negatively impact quality of life and may contraindicate DBS treatment, and early identification is therefore important to allow necessary time for rehabilitation before the surgery. These aspects are assessed using screening tests, such as the Mini-Mental State Examination (MMSE), and complemented by specific tests that examine different components of cognitive ability, including executive function, memory, attention, and verbal fluency.^10,11^

Given the importance of conducting neuropsychological assessments in PD patients who are candidates for DBS surgery, the primary objective of this study was to determine the neuropsychological profiles (NP) of patients treated at the outpatient movement disorder clinic of the ISCMPA and to explore the association between neuropsychological scores and age at onset of disease, disease duration, degree of motor involvement, quality of life, sleep quality and other variables.

## METHODS

A cross-sectional study was conducted. All literate Parkinson's patients receiving treatment at the Movement Disorder Clinic of the ISCMPA in Porto Alegre between August 2013 and August 2014 were asked to participate in the research project. The inclusion criteria encompassed PD patients who had initially been ruled out for dementia diagnosis through an MMSE assessment using mean scores adjusted by educational level: 25 points for individuals with 1-4 years of education; 26.5 points for 5-8 years; 28 points for 9-11 years; and 29 points for more than 11 years of education.^12^ The sample comprised 30 PD patients being followed at the same outpatient clinic, awaiting DBS surgery, previously screened by the neurology department. All patients were assessed during the "on" phase of medication.

**Instruments.** All instruments were used according to the Movement Disorders Society (MDS) recommendations and can be described as follows:

*Montreal Cognitive Assessment (MoCA):^13^* This instrument assesses eight different cognitive domains. Included in the set of items that constitute this tool are five of the six tasks most frequently used to screen for dementia. The Brazilian version of the MoCA is a valid and reliable instrument for screening for mild cognitive impairment among older individuals, validated by Memoria, 2012.^14^

*Frontal Assessment Battery (FAB):* This is a screening tool to assess frontal executive functions (both executive and motor components) in a single instrument. The test has good psychometric properties and construct/criteria validity, according to a study by De Paula, 2013.^15^

*Beck Depression Inventory (BDI):^16^* A self-assessment measurement of mood widely used in both research and clinical settings. The depression intensity classification has the following range : minimal depression (0-9), mild depression (10-16), moderate depression (17-29) and severe depression (30-63). Validated in Brazil by Cunha, 2001.

*Verbal Fluency Test (Semantic Category):^17^* Checks for the existence of semantic memory loss and impairment of search strategies related to executive function. The test was validated for the Brazilian population by Brucki, 2004.

*Parkinson's Disease Questionnaire-39 (PDQ-39):^18^* This has been indicated as the most appropriate instrument for assessing the QoL of PD patients and is sufficiently robust to be used in trans-cultural studies. It assesses eight different domains: mobility, daily activities, emotional well-being, stigma, social support, cognition, communication and bodily discomfort. It was adapted to Brazilian Portuguese by the Health Services Research Unit (Department of Public Health and Primary Care - University of Oxford) in 2005.

*Parkinson's Disease Sleep Scale (PDSS):^19^* This is a 15-item scale assessing a variety of sleep disorders commonly associated with PD. The instrument has been translated and validated internationally and its validation was performed in Brazil by Margis, 2010.

*Unified Parkinson's Disease Rating Scale revised by the Movement Disorder Society (UPDRS- MDS/2008):* The UPDRS was created in 1987, revised and expanded in 2008, where the UPDRS- MDS/2008 has demonstrated high consistency both internally and correlated with the original UPDRS (ρ=0.96).^20^ It is a gold-standard tool for assessing the disease's symptoms as well as its impairments in research and clinical environments.

*Hoehn and Yahr Scale of Degree of Disability:^21^* This scale was developed in 1967. It is fast and practical for indicating a patient's general state. A modified version of the H&Y scale was developed more recently to include intermediate stages, and was used in this study. The H&Y scale is widely used and accepted for assessing the stages of PD.

**Procedures.** Patients who met the inclusion criteria were invited to participate in the study after consulting with a neurologist at the outpatient clinic. Neuropsychological data was collected at the ISCMPA Movement Disorder Clinic in a single session. All patients were assessed in the morning while they were in their optimal "on" state. The tests were performed in a spiraled order to avoid response bias and applied by researchers from the research group who had been previously trained for this assessment. The assessments performed indicated the neuropsychological profiles of the clinic patients and enabled the professionals involved to monitor the clinical evolution and surgical indication for each case. All individuals who participated in the study signed an informed consent form developed according to the criteria of the Research Ethics Committees of the UFCSPA and ISCMPA, where the research project was approved under CAAE 17123013.6.0000.5335.

**Statistical analysis.** Descriptive and inferential statistical analyses were performed to characterize the sample. The descriptive analyses were expressed as mean, standard deviation (SD) and absolute and relative frequencies. The tests that had normal distributions were analyzed using Pearson's correlation and results expressed as mean (SD). Data with non-normal distributions were analyzed using Spearman's correlation and their results were expressed as median (25^th^ and 75^th^ percentiles). The Correlation Tests were used because the aim of the coefficient is to identify the correlation between the two variables. To compare means, the *t*-test was used to assess whether or not characteristic symptoms of that dimension were present. The SPSS v.21.0 statistical program was employed and the significance level for all tests was p≤0.05.

## RESULTS

Thirty PD patients were assessed at the ISCMPA Movement Disorder Clinic: 20 males (66.7%) and 10 females (33.3%) aged 28-77 years. Other sample characteristics are shown in Table 1.

**Table 1:** Sample characteristics.

Characteristic		n=30
Age (years) - mean±SD		59.7±10.7
Gender - n(%)	Male	20 (66.7)
	Female	10 (33.3)
Age at disease onset (years) - mean±SD		50.4±11.1
Duration of PD (years) - mean±SD		9.33±4.08
Years of education - md (P25 - P75)		4 (4-10)
Hoehn & Yahr - n(%)	1	1 (3.3)
	2	23 (76.7)
	3	6 (20.0)
MDS-UPDRS - md (P25 - P75)		29 (22-50)*

SD: standard deviation; n: sample size; md: median; PD: Parkinson’s disease; MDS-UPDRS: Movement Disorder Society - Unified Parkinson’s Disease Rating Scale.

* n: 26.

Among the disease's leading symptom, tremor was the most frequent (80% of the cases) while rigidity or bradykinesia were reported in 20% of the cases. Resting tremor was reported as a current symptom by 53.3% of the patients while rigidity or bradykinesia were reported by 46.7%. The mean staging of the disease using the H&Y scale was 2.17 (SD 0.46), with scores ranging from 1 to 3 (mild to moderate).

Section III of the MDS-UPDRS was concluded by 26 of the 30 patients and the median score for the 26 assessed patients was 29 points (25^th^ to 75^th^ percentiles: 22 to 50).

On a scale ranging from 0 to 100, the median (mean of 35.33%, SD 17.06) quality of life perceived by the patients was 31.5 (21.5 to 46.5) with highest scores related to mobility (23%) and ADL (28%) domains, indicating greater discomfort in these aspects. Mean perceived sleep was 99.3 (SD 31.11) on a scale ranging from 0 to 150, indicating moderate-to-good sleep.

Frequency and severity of symptoms on the neuropsychological tests revealed impairment of frontal functions in 56.7% of the patients as measured by the FAB and cognitive deficits in 76.7% of the patients on the MoCA. The patients exhibited low semantic verbal fluency on BDI scores in 30% of cases. On the mood assessments, 53.3% of the patients had no depression or had minimal depression, while 30% had mild-to-moderate depression, 13.3% had moderate-to-severe depression and 3.3% had severe depression. Data for each sub-item are shown in Table 2.

**Table 2:** Cognitive assessment.

Cognitive aspects	n=30
FAB - mean±SD	
Conceptualization (0-3)	2.5±0.6
Mental Flexibility (0-3)	2.0±0.9
Motor Programming (0-3)	2.7±0.5
Sensitivity to Interference (0-3)	1.9±0.9
Inhibitory Control (0-3)	1.6±1.1
Environmental Autonomy (0-3)	2.8±0.4
Total	13.6±3.4
FAB Classification - n(%)	
<16 (with cognitive impairment)	17 (56.7)
≥16 (without cognitive impairment)	13 (43.3)
MoCA - mean±SD	
Visuospatial/Executive Function (0-5)	2.3±1.5
Naming (0-3)	2.7±0.7
Attention (0-6)	4.6±1.6
Language (0-3)	1.5±0.9
Abstraction (0-2)	1.2±0.6
Delayed Recall (0-5)	2.6±1.4
Orientation (0-6)	5.9±0.3
Total	22.0±4.8
MoCA Classification - n(%)	
<26 (with cognitive impairment)	23 (76.7)
≥26 (without cognitive impairment)	7 (23.3)
Semantic VF - mean±SD	12.0±4.3
Semantic VF Classification - n(%)	
Low fluency	9 (30.0)
Normal fluency	21 (70.0)

SD: standard deviation; n: sample sizer; FAB: Frontal Assessment Battery; MoCA: Montreal Cognitive Assessment; VF: Verbal fluency.

The correlation between age at disease onset and neuropsychological assessment scores showed statistically significant results on the FAB tests for total score (r=–0.463, p≤0.01) and for the sub-items related to phonemic verbal fluency (r=–0.511, p≤0.00), motor programming (r=–0.404, p≤0.02) and inhibitory control (r=–0.429, p≤0.01). Similarly, a significant correlation was detected between the variables cited above on the results for the MoCA total score (r=–0.437, p≤0.01) and its sub-items related to executive and visuo-spatial functions (r=–0.435, p≤0.01), as well as for semantic verbal fluency score (r=–0.505, p≤ 0.00).

The disease duration and staging only showed significant results for the MoCA naming sub-item, with r=–0.469, p≤ 0.00 for duration and r_s_= –0.584, p≤0.00 for severity on the H&Y scale. The quality of life questionnaire mobility domain showed a negative correlation with total FAB (r_s_= –0.393, p≤0.03), the FAB inhibitory control sub-item (r_s_= –0.395, p≤0.03), total MoCA (r_s_= –0.448, p≤0.01) and the MoCA attention sub-item (r_s_= –0.509, p≤0.00). In the stigma domain, there was a correlation with the FAB motor programming sub-item (r_s_= –0.424, p≤0.02). For the communication domain, there was a correlation with the MoCA executive and visual-spatial functions sub-item (r_s_= –0.363, p≤0.04).

**Figure 1:**
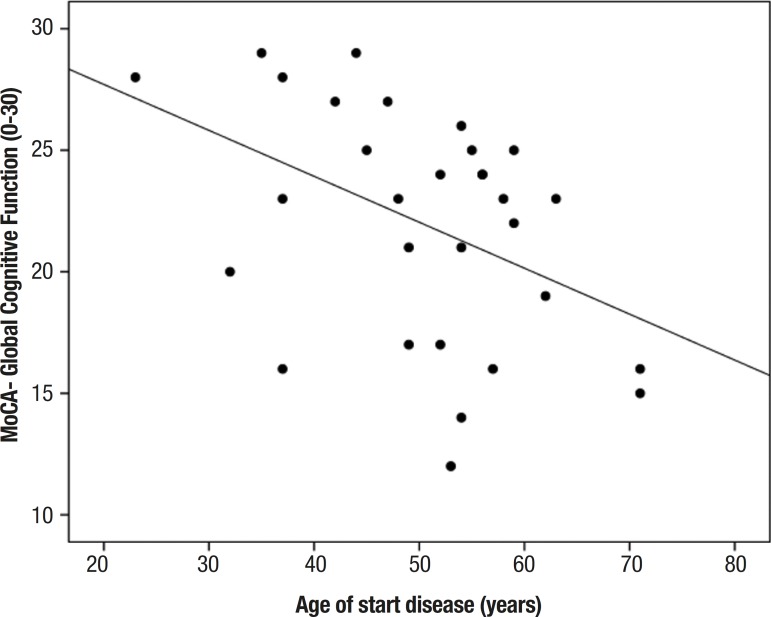
Correlation between total score on the Montreal Cognitive Assessment (MoCa) and age at disease onset (years).

**Figure 2:**
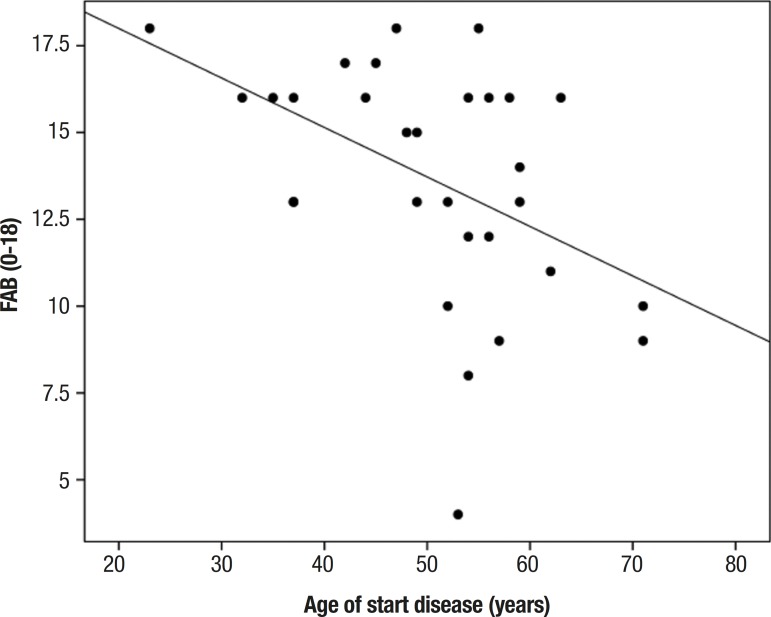
Correlation between total score on the Frontal Assessment Battery (FAB) and age at disease onset (years).

The analysis included assessment of correlations between MoCA (final score) and age (r_s_= –0.553, p≤0.00) or education level (r_s_= –0.602, p≤0.00); FAB (final score) and age (r_s_= –0.545, p≤0.00) or education level (r_s_= –0.452, p≤0.01).

As expected, all of the results described above demonstrate a moderately strong correlation among the variables. Correlations between NP findings and sleep quality, current rigid or tremor symptom, or initial tremor symptom were not statistically significant.

## DISCUSSION

DBS surgery is indicated in specific cases and can benefit a small percentage of PD patients. Many studies have shown that this type of surgical treatment can significantly improve the motor condition of individuals with fluctuations and dyskinesias.^8,22,23^

Selection criteria include: an established PD diagnosis for at least five years; motor complications; unsatisfactory control or intolerance to drug treatment; age (since younger patients and those with the disease for less time show the most beneficial effects); and absence of dementia and severe depression.^6,24^ The subjects who comprised this study's sample had demographic characteristics that were compatible with these criteria. They were patients in the initial stages of the disease, as shown by the H&Y scale; they had motor symptoms that impaired their mobility and daily activities, as evidenced by the PDQ-39; and most of the group had minimal or no depression.

However, over half of the patients assessed had cognitive symptoms (on both the FAB and the MoCA). The MoCA scores indicated cognitive impairment in 76.7% of the subjects. The most affected constructs were related to executive functions, supporting the findings of international studies^25,26^ in which persons with PD had impaired executive functions even in the initial stages of the disease. In the Elgh, 2009 study, 30% of the subjects had cognitive deficits in the executive function domain and 16% had impairments in two or more domains. All of these patients were at the initial stage of the disease.^25^ Regarding Brazil, Campos-Souza compared PD patients with subjects without the disease. The results showed worse performance for the experimental group compared to the control group. On the Stroop test for inhibitory control, the control group had a mean score of 35.83 compared to 24.2 for the PD1 group. For visual-spatial construction, the control group had an average score of 30.84 while the PD2 group scored 26.2, as measured by the Rey Complex Figure Test. Results on the Wisconsin Card Sorting Test showed statistically significant differences between the group mean scores for cognitive flexibility (CG= 27.50, PD1= 46.57 and PD2= 44.14). Nevertheless, few Brazilian studies have investigated PD executive dysfunctions.^27^

It has been suggested that executive performance depends on the integrity of the prefrontal cortex and other related structures. Individuals with PD have less deactivation of the cortex and have shown an inverse pattern of activation and deactivation, accounting for their poor functioning specifically during executive function tasks. PD patients had low scores for attention and inhibitory control (which are included in these functions) especially on the FAB, which measures competency in prioritizing a given item among a large number of simultaneous stimuli. These attention processes assist, enhance or inhibit other neuropsychological processes (such as memory, perception and language) to enable a task to be performed effectively.^28^

The fact that this study's results showed such a high percentage of cognitive dysfunction points to the need for comprehensive and thorough assessments in the preoperative period because subjects screened for surgical assessment using tools such as the MMSE may present impairment in executive functions, language and memory that are not detected by the test. In addition, interventions can be introduced to maintain or improve this situation—namely neuropsychological rehabilitation and training tasks, which have only been studied recently and have received little attention nationally.^29^

Also in relation to this study's results, the moderate correlation found between the neuropsychological scores and age at disease onset points to the important discussion about the ideal time for performing the surgical procedure. As this study has shown, cognitive changes occur in older individuals, although most patients have some cognitive deficit not meeting the criteria for dementia or mild cognitive impairment (MCI) established by the MDS. Missing the right time window could contraindicate the procedure because of the emergence of cognitive complications, where cognitive impairment is one of the exclusion criteria and it remains unclear whether this deficit is a predictor of dementia in the postoperative period.

It is currently being discussed whether to bring forward the time window for indicating DBS surgery in order to target younger patients with earlier disease onset and less time with the disease. The main goal would be to maintain the patient's quality of life before motor and cognitive complications interfere with social aspects and activities of daily living or even contraindicate the surgery (in cases of cognitive impairment).^5,30^ One limitation of this study is the small number of subjects comprising the sample, as the surgical indication is meticulous and includes specific cases. Another limitation is the absence of a control group matched for age and time with PD. This control pairing was considered but deemed unfeasible. The small sample and mild or moderate cases hamper grouping because only a few cases would be allocated to each group. This precludes comparison of means or logistic regression.

In conclusion, in recent years, interest in PD's non-motor symptoms has been evidenced by a growing body of research on the subject. With specific regard to neuropsychological factors, questions remain about which domains are most affected, whether there are better prognosis predictors and what effect DBS has on cognitive symptoms. In this context, the present study contributes to expanding results based on scientific evidence.

Furthermore, this study has described the neuropsychological profile of patients who are candidates for DBS surgery, allowing clinical staff to assess the best clinical procedure to treat the disease in the pre-surgical period. This information will also serve as a control for monitoring disease evolution and cognitive deterioration. This information is especially relevant for patients who will undergo the surgical procedure, as they will have a baseline against which to assess their postoperative condition.

Finally, this study points to the relevance of investigating PD's non-motor (especially cognitive) aspects and we propose a discussion of rehabilitation strategies linked to the indication of surgery at the most beneficial time for the patient. This assessment should be expanded to other movement disorder centers using the control group as a basis for comparing neuropsychological findings.
